# Infliximab’s influence on anastomotic strength and degree of inflammation in intestinal surgery in a rabbit model

**DOI:** 10.1186/1471-2482-14-23

**Published:** 2014-04-24

**Authors:** Erik Frostberg, Petter Ström, Oke Gerke, Niels Qvist

**Affiliations:** 1 Surgical Department A, Odense University Hospital, Sdr. Boulevard 29, 5000 Odense, C, Denmark; 2 Department of Nuclear Medicine, Odense University Hospital, Odense, Denmark & Department of Business and Economics, Centre of Health Economics Research, University of Southern Denmark, Odense, Denmark

**Keywords:** Infliximab, Intestinal anastomosis, Rabbits, Tensile strength, Wound healing

## Abstract

**Background:**

Infliximab, a TNF-α inhibitor, is a potent anti-inflammatory drug in the treatment of inflammatory bowel diseases. Recent studies have investigated the effect of infliximab treatment on postoperative complications such as anastomotic leakage, however, with conflicting results and conclusions. The purpose of this study was to investigate whether a single dose infliximab has an adverse effect on the anastomotic healing process, observed as reduced anastomotic breaking strength and histopathologically verified lower grade of inflammatory response, in the small intestine of a rabbit.

**Methods:**

Thirty New Zealand rabbits (median weight 2.5 kg) were allocated to treatment with an intravenous bolus of either 10 mg/kg infliximab (n = 15) or placebo (n = 15). One week later all rabbits underwent two separate end-to-end anastomoses in the jejunum under general anesthesia. At postoperative day three, the anastomotic breaking strength was determined and histopathological changes were examined.

**Results:**

The mean value of anastomotic breaking strength in the placebo group was 1.89 ± 0.36 N and the corresponding value was 1.81 ± 0.33 N in the infliximab treated rabbits. There was no statistically significant difference between the groups (p = 0.51). The infliximab-treated rabbits had a significant lower degree of inflammatory infiltration response compared to the placebo group (p = 0.047).

**Conclusions:**

Our conclusion, limited by the small sample sizes in both groups, is that a single dose of infliximab, given one week prior to surgery, does not have an impact on the anastomotic breaking strength on the third postoperative day in the small intestine of rabbits.

## Background

Numerous systemic and local factors play a central role in the healing of intestinal anastomoses [1]. Important factors in extracellular matrix are collagen fibers, fibroblasts and immune cells that regulate wound strength in the early postoperative healing process [2,3]. In the normal inflammatory response, monocytes migrate into the wound where they are transformed into macrophages. The tumour necrosis factor-α (TNF-α) molecule is primarily released by the macrophages and since TNF-α stimulate the collagenase production, inhibit collagen synthesis and increase fibroblast proliferation and production of interleukin-6 and prostaglandin E_2_, it is considered to be one of many important chemical inflammatory mediators essential for tissue remodelling and wound healing [4,5]. Infliximab (IFX) is a chimeric anti-tumour necrosis factor monoclonal antibody that binds to TNF-α molecules with high affinity. Due to this mechanism, it effectively inhibits the TNF-α molecule’s multiple biological properties related to inflammation and proliferation [6].

IFX has shown to be effective in the treatment of patients with inflammatory bowel disease, such as Crohn’s disease [7-9] and ulcerative colitis [10,11]. However, due to treatment failure many of these patients still require surgery [12-14]. Some studies have shown an increased risk of anastomotic leakage and other postoperative complications in patients preoperatively treated with IFX [15-17], and other studies have found no increased risk [18-22]. The discrepancy between these results could be due to different study designs and patient material. Two recent experimental animal studies, with intestinal anastomoses in rats, suggest that TNF-α inhibitor do not have a significant impact on the anastomotic healing process, measured by anastomotic breaking strength and low TNF-α levels in blood and tissue samples [23,24]. However, it is suggested that the biological properties of IFX may have other possible effects on anastomotic wound healing [24] and therefore further research regarding possible effect of IFX on the intestinal healing process is warranted.

The purpose of this study was to investigate whether a single dose IFX has an adverse effect on the anastomotic healing process, observed as reduced tensile strength and histopathologically verified lower grade of inflammatory response, in the small intestine of a rabbit.

## Methods

### Animals

Thirty female New Zealand rabbits, three months old and with a median weight of 2.5 kg (range 2.0 to 3.1 kg) were included. Female rabbits were used throughout the study to avoid gender as a potential confounding factor, although gender is not a factor in the healing of small intestinal anastomoses [25]. All rabbits were housed in wire cages with a shelter and plateau, including wood shavings and hay. They were kept at room temperature (18–20°C) with 40-60% humidity and a 12-hour light/dark cycle. All rabbits had access to standard laboratory diet and water as needed, and they were acclimatized for seven days before surgery.

### Study design

The animals were randomized to treatment with IFX (n = 15) or placebo (n = 15). The treatment group received a single bolus (10 mg/kg) of infliximab (REMICADE, Centocor, Leiden, Holland) intravenously, and the placebo group received the same volume of isotonic saline. The rabbits underwent surgery one week after medication and were sacrificed at the third postoperative day (POD3) based on the results (unpublished) from a pilot study, where we found a rupture in the anastomotic line in 66% of the anastomosis on POD3 and in only 14% of the anastomosis on POD7. Our interpretation was, that the anastomosis was weaker in the POD3 group, which is supported by the fact that at this time the inflammatory reaction in wound healing is high and gradually replaced by the proliferating phase. In addition to this, it is well known that anastomotic leakage most often develop from POD3 to POD5. The exact time course of strength development in the rabbit small intestine is to our knowledge not available in the literature.

### Surgical procedures

All rabbits were sedated with a 0.3 ml/kg mixture of fentanyl citrate 0.315 mg/ml and fluanisone 10 mg/ml (HYPNORM, Vetpharma, Leeds, United Kingdom) administered subcutaneously together with 2 mg/kg of midazolam (DORMICUM, 5 mg/ml; Roche, Basel, Schweiz). In addition 10 mg/kg of propofol (PROPOVET, 10 mg/ml, Abbott Laboratories Ltd, Berkshire, Great Britain) was given intravenously. After sedation, all rabbits were intubated with a size 3.5 endotracheal tube (Rüsch, Kernen, Germany). The animals were anesthetized with 4 vol.% sevoflurane (SEVOFLURANE, Abbott Scandinavia AB, Solna, Sweden) via a MCM 801-ventilator (Dameca, Rødovre, Denmark). For perioperative analgesia, fentanyl (HALDID, 50 μg/ml, Janssen-Cilag, Beerse, Belgium) was administered intravenously at a rate of 5 ml/hour using an infusion pump (Terumo STC-526, New Jersey, USA). The rabbits were monitored by a pulse oximeter (NONIN 8500 V, Nonin Medical, Minnesota, USA).

Under aseptic conditions, a 4 cm midline laparotomy was made and the *extremitas appendices vermiformis caeci* was identified. Two separate end-to-end anastomoses were made, approximately 25 and 50 cm proximal to the appendix. All anastomoses were made with interrupted inverted single-layer 5–0 non-absorbable sutures (PROLENE, Ethicon, Johnson & Johnson Nordic, Birkerød, Denmark). The musculofascial layer was closed with interrupted 3–0 absorbable sutures (VICRYL, Ethicon, Johnson & Johnson Nordic, Birkerød, Denmark) and the skin with a continuous 4–0 non-absorbable suture intracutaneously (ETHILON, Ethicon, Johnson & Johnson Nordic, Birkerød, Denmark). Prior to skin incision 0.2 ml/kg of a mixture of sulfadoxin 200 mg/ml and trimethoprim 40 mg/ml (DUOPRIM VET, Intervet International B.V, Boxmeer, Holland) was given intravenously and 5 ml of isotonic saline were administrated subcutaneously after the surgery.

At POD3, the rabbits were euthanized with an overdose of 2 ml intravenous pentobarbital (PENTOBARBITAL, 200 mg/ml; KU Life, Copenhagen, Denmark). A relaparotomy was performed and the two anastomoses were identified and carefully freed from adhesions. The anastomoses were resected with a 2 cm margin on each side, and cleaned for fecal contents with saline. The sutures were left in place. The proximal anastomosis was used to test anastomotic breaking strength and the distal anastomosis to histopathological analysis.

The choice of performing the anastomoses in the small intestine was justified by the fact that the majority of intestinal resections in inflammatory bowel disease involve small intestines and/or proximal colon. Another important aspect was that, that the anatomy of the rabbit colon is significant different from humans and anastomosis on/to the colon would necessitate extensive mobilization of the colon with increased risk of postoperative morbidity, which could be a confounding factor.

### Anastomotic breaking strength

The proximal anastomosis was mounted, with 20 mm between the clamps and with the anastomosis in the middle, in a testing machine (LF Plus; Lloyds Instruments, Fareham, UK) equipped with an XLC 10 N load cell (Lloyds Instruments, Fareham, UK). The intestine was stretched at a constant deformation rate of 10 mm/min. The anastomotic breaking strength, defined as the minimal strength necessary to rupture the anastomosis, was derived from the load-strain curve calculated by the software (Nexygen, Lloyds Instruments, Fareham, UK). The site of rupture was noted as either; located in the anastomosis or outside the anastomotic line.

Although the specimen was mounted in the machine with precise accuracy, there always was the uncertainty about the tension test machine to apply an equally distractive force to the entire circumference in each specimen. To minimize this possible error, we programmed the test to start recording after the specimen had been subjected to a tension of 0.1 N. By doing this, we had the opportunity to detect signs on distraction, and we could interrupt the test, remount the specimen and restart if necessary.

### Histopathological analysis

A sample of the distal anastomosis with the sutures *in situ* was fixed in 4% formaldehyde. The sample was dehydrated and embedded in paraffin blocks and sliced 3 μm thin perpendicular to the anastomotic line. Staining was made with hematoxylin and eosin. A conventional binocular Leica DMR (Leica Microsystems A/S, Herlev, Denmark) light microscope with objective 40/0.75 was used. The area within two millimetres related to the anastomotic line was examined and the grade of inflammatory infiltration response in the anastomotic line was scored using a four-graded scale, with a 0–3 point value; absent (0 points), slight (1 point), mild (2 points) and intense (3 points). The examiner of the histological specimens was blinded with regard to treatment group to avoid bias.

### Statistical analysis

Due to the lack of a priori information for sample size calculations, we conducted an interim analysis in order to adjust for the final sample size. Results from that interim analysis, based on 6 rabbits treated with IFX, suggested the anastomotic breaking strength on small intestinal anastomosis to be 1.61 N with a standard deviation of 0.3. Assuming a true, clinical significant difference of 20% between the groups with respect to anastomotic breaking strength, 14 rabbits in each group were sufficient to show a statistical significant difference between the groups with a power of 80% at a significance level of 5% (two-sided). Drop-outs were not replaced due to time constraints.

The data was analyzed according to type, i.e., categorical variables were presented as frequencies and corresponding percentages and continuous variables by mean ± standard deviation (SD). The assumption of a normal distribution of data was assessed visually by means of a quantile-quantile plot for the respective variable. Student’s t-test with unequal variances was applied to compare the weight loss between the groups. Mann–Whitney U test (Wilcoxon rank-sum test) was performed to compare the weight, anastomotic breaking strength and number of sutures between the groups. Fisher’s exact test (two-sided) was used to test the site of rupture and to compare the histological parameters between the groups. P-values <0.05 were considered statistically significant without adjustment for multiple testing. The sample size calculation and statistical analysis was performed using Stata/IC 11.0 (StataCorp LP, College Station, Texas, USA).

### Ethical considerations

The project was performed at the central animal facility of the University of Southern Denmark, and a approval (No. 2005/561 - 1066) to perform the project was granted by the Danish Animal inspectorate on April 15th, 2010.

## Results

In total, three rabbits died perioperatively, one in the IFX-treated group and two in the placebo group, due to anesthetic complications. Their data were excluded from the study. The study was completed with fourteen rabbits treated with IFX (n = 14) and thirteen rabbits in the placebo group (n = 13). Descriptive statistics on the rabbits included are shown in Table 1.

**Table 1:** Descriptive statistics on the rabbits included in the study

**Parameter**	**Placebo (N = 13)**	**Treatment (N = 14)**	***p*-value**
**Number of sutures**			
Mean	14.5	14.5	0.80*
**Anastomotic breaking strength (N)**			
Mean	1.89	1.81	0.51*
Std. dev	0.36	0.33	
Minimum	1.34	1.32	
Maximum	2.46	2.31	

*Mann–Whitney U test (Wilcoxon rank-sum test).

A decrease in weight at euthanasia compared to the preoperative weight was found in both groups (IFX-treatment -8.1%; placebo: -7.7%). There was no difference in weight loss between the groups (p = 0.66). In none of the animals an increase in body weight was observed.

### Anastomotic breaking strength

**Figure 1:**
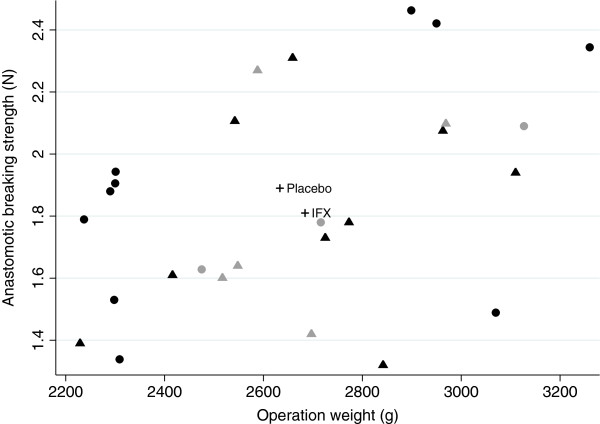
**Correlation between anastomotic breaking strength, measured in Newton (N), and operation weight, measured in gram (g).** ● = rabbit treated with placebo, ▲ = rabbit treated with infliximab, + = mean values of anastomotic breaking strength and operation weight. Symbols in gray are anastomoses that ruptured outside the anastomotic line.

**Figure 2:**
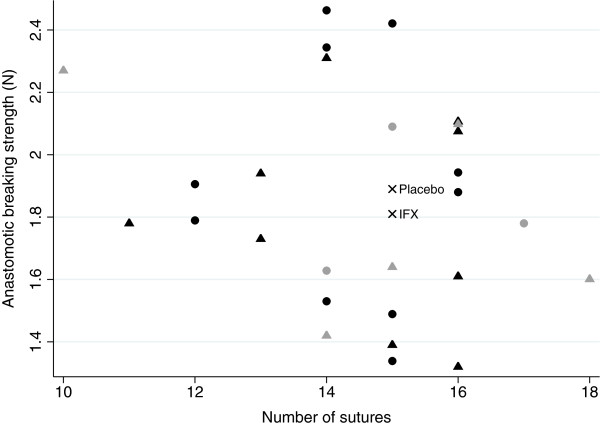
**Correlation between anastomotic breaking strength, measured in Newton (N), and numbers of sutures in the anastomosis.** ● = rabbit treated with placebo, ▲ = rabbit treated with infliximab, × = mean values of anastomotic breaking strength and median value of number of sutures. Symbols in gray are anastomoses that ruptured outside the anastomotic line.

The mean value of anastomotic breaking strength in the placebo group was 1.89 ± 0.36 N and the corresponding value was 1.81 ± 0.33 N in the IFX-treated rabbits. There was no statistically significant difference between the groups (p = 0.51) (Table 1). The site of rupture was within the anastomotic line in nineteen out of twenty-seven (70%) rabbits, and it was similar in the two groups. However, 30% of the anastomosis ruptured outside the anastomosis, which is illustrated in Figures 1 and 2. The figures shows that five of the eight anastomoses that ruptured outside the anastomosis had 15 or fewer sutures, and that three of the eight anastomoses had 16 or more sutures. The median number sutures used in the anastomoses where 15. Furthermore, the figures also presents that five of the anastomoses that ruptured outside the anastomotic line was in IFX-treated rabbits, and three in the placebo group. A supplementary analysis with these eight animals excluded was done, and it did not have any impact on the results. It showed only a marginal increase in anastomotic breaking strength in the placebo-group with 0.02 N, and did not affect the mean in the treatment group or the p-value (data not shown). The use of different number of sutures in the anastomoses, which also is seen in other animal studies [26-28], was necessary to avoid anastomotic leakage since the intestine diameters varied in size. No difference in the use of number of sutures per anastomosis between the groups was observed.

### Histopathological analysis

**Figure 3:**
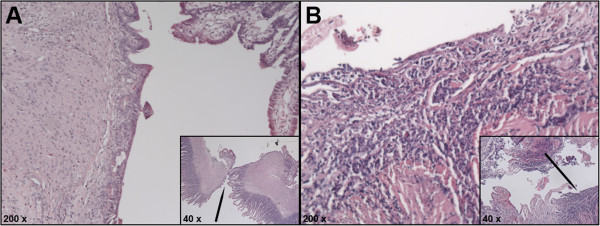
**Histological images depicting the inflammatory infiltration response in the anastomotic site.** Depicts the inflammatory infiltration response in the anastomotic site evaluated with a four-graded scale. The inserted images show the anastomotic site at magnification of approximately 40 x and the black arrow points at the magnified area (approximately 200 x). **(A)**. Slight inflammatory infiltration response in a rabbit from the control group. **(B)**. Intense inflammatory infiltration response in a rabbit from the control group.

Eleven rabbits in the placebo-group (84.6%) were scored with two or more points (2 points: 23.1% and 3 points: 61.5%), and nine rabbits (64.3%) in the IFX-group (2 points: 50.0% and 3 points: 14.3%). Only two rabbits in the placebo group scored one point (15.4%), compared to five (35.7%) in the IFX-group. No rabbits were assessed with zero points. Thus, the placebo group had a significant higher grade of inflammatory infiltration response in the anastomotic site compared to the IFX-treated rabbits (p = 0.047) (Table 2). The visual difference between slight and intense inflammatory infiltration is depicted in Figure 3A and B.

**Table 2:** Histological scores for each group, examined as the overall inflammatory infiltration response in the anastomotic site, presented as categorical data with fraction in column percentage

**Score**	**Placebo (N = 13)**	**Treatment (N = 14)**
0	0 (0.0%)	0 (0.0%)
1	2 (15.4%)	5 (35.7%)
2	3 (23.1%)	7 (50.0%)
3	8 (61.5%)	2 (14.3%)
Total	13 (100%)	14 (100%)

Two-sided Fischer’s exact test shows that the placebo-group has a significant higher score (p = 0.047).

## Discussion

The inflammatory reaction plays an important role in wound healing [1,2,4,5], and since IFX has an anti-inflammatory effect this could be one possible explanation to the increased risk of anastomotic leakage that has been reported in humans treated with IFX prior to surgery. In the study, we found a significant lower degree of inflammatory infiltration in the treatment group compared to the placebo group on POD3, but there was no significant difference in anastomotic breaking strength between the groups.

The ideal postoperative day for investigating anastomotic strength has been debated, and no consensus has been obtained. Thompsen et al. found that the healing process at POD3 changes from the inflammatory reaction to the proliferation phase [4], which in theory might be the most critical time for wound dehiscence. Christensen et al. investigated anastomotic rupture in rats at postoperative day 2, 4 and 6 using the bursting pressure technique. They found that 60% of the tested segments ruptured outside the anastomosis at day 6 [29]. Although we used a different technique than Christensen et al., our results were in line with their findings since most of the anastomoses in our study ruptured in the anastomotic line at POD3 without any differences between the two groups.

To analyze the physical strength of the anastomosis, two main methods can be used: bursting pressure and the tension test [3]. Ikeuchi et al. concluded that tension test, evaluated as minimal intestinal strength, is a better method to evaluate anastomotic healing, and that bursting pressure can evaluate the overall anastomotic integrity but may reflect anastomotic healing less accurately [26].

The overall inflammatory infiltration response in the anastomotic line was graded according to a four-graded scale. In reflection, and since TNF-α mainly is produced by macrophages, an estimation of these cells in the anastomotic site may have been interesting. Immunohistochemical staining specific for TNF-α, as a complement to hematoxylin and eosin, should preferably be used to recognize the macrophages because they are difficult to distinguish from fibroblasts in HE-staining.

Ågren et al. aimed to investigate whether an increase in matrix metalloproteinase (MMP) affects the anastomotic dehiscence in rats, and since TNF-α induces the expression of MMP activity the hypothesis was that TNF-α indirectly might influence the pathogenesis of the anastomotic healing process. The authors found that the use of TNF-α inhibitor (p38 MAP kinase inhibitor), given one hour before anastomotic surgery and then on POD 1 and 2, prevented increased levels of TNF-α in the anastomotic wound. However, a significant difference in anastomotic breaking strength was not detected between the groups [23]. This is in line with our findings, since our results showed that the IFX group had a significant lower grade of inflammatory response in the anastomotic site, which could be explained by locally decreased levels of TNF-α. In addition to this, inhibition of the TNF-α molecule's properties, which as one of many chemical inflammatory mediators promote leukocyte-endothelial adhesions as a prelude to leukocyte emigration from the vessels into the wound, can explain our histological findings of lower grade of inflammatory response.

Iglesias et al. tested the effect of another TNF-α inhibitor (etanercept) in cutaneous wound healing in mice, and they found no significant difference in wound healing time (measured over 20 days), morphological changes or re-epithelialization rate between the treated and untreated group. As in our study, they found that the inflammatory cell counts in the wound area were higher in the non-treated animals although this was not statistically significant. They concluded that biological therapies, that inhibit the effect of the TNF-α molecule, does not affect wound healing and does not need to be suspended preoperatively [30].

Zubaidi et al., investigated the overall expression of cytokines in rat intestinal, facial and cutaneous wound healing. Interestingly they found that local expression of TNF-α on POD1, after intestinal anastomotic surgery, significantly decreased and sustained decreased over 14 days during the postoperative period. A similar result was found in colonic anastomosis, while increased levels of TNF-α in cutaneous and facial wounds was observed [24]. These finding may have clinical importance, since it indicates that inhibition of TNF-α probably not affects the anastomotic healing process, as a result of the already low levels of TNF-α in the wound area. However, they did not particularly test the influence of TNF-α inhibition in wound healing, therefore further research is required to elucidate this. With this in mind, it would be very interesting to measure the serum and local anastomotic levels of TNF-α. In another study where the effect of IFX on neuronal apoptosis was examined in rabbits, a significant reduction (approx. 50%) in the blood concentration of TNF-α was found two weeks after subcutaneous administration of a dose of 5 mg/kg [31] and in a recent study on another TNF-α inhibitor (adalimumab), the serum concentration in rabbits was within the recommended level after repeated administration according to clinical use [32]. Since we aimed to test IFX possible influence on the intestinal healing, the treatment group received the double of therapeutic dose recommended in humans with inflammatory bowel diseases. Measuring the levels of TNF-α in a venous blood sample could have been used to validate the IFX treatment in this study, but there are no reasons to believe, that the pharmacodynamics of IFX in rabbits differ from humans.

At last, it is also important to mention that the comparably small sample sizes of 13 and 14 rabbits in our groups are only sufficient to show comparably large effects. Small differences as seen in our study can only be confirmed statistically by larger studies.

The present study used a single dose of IFX one week prior to surgery. A long-term course of IFX given preoperatively to patients with severe symptoms of inflammatory bowel disease is common. In addition to this, we used a healthy rabbit intestine, where wound healing may differ from that seen in bowels affected with Chron’s disease or ulcerative colitis. These facts are important shortcomings in the comparison of the present results with the clinical situation. We suggest that additional studies with a long-time preoperative treatment with IFX should be done to investigate whether IFX may have a possible detrimental effect on the anastomotic healing process.

## Conclusion

Our conclusion, limited by the small sample sizes in both groups, is that a single dose of IFX, given one week prior to surgery, does not have an impact on the anastomotic breaking strength on the third postoperative day in the small intestine of rabbits.

## Competing interest

The authors declare that they have no competing interests.

## Authors’ contributions

EF carried out the study design and research protocol, performed the surgical procedures, histological examination and measuring of breaking strength, executed the statistical part of the project and drafted the manuscript. PS contributed to the design of the study and research protocol, and participated in the surgical and histological procedures and the measuring of anastomotic breaking strength. OG helped with the statistical analysis and contributed to the discussion. NQ participated in the design of the study and research protocol and helped to draft the manuscript. All authors have read and approved the final manuscript.

## Pre-publication history

The pre-publication history for this paper can be accessed here:

http://www.biomedcentral.com/1471-2482/14/23/prepub
